# Potential use of serum based quantitative real-time PCR for the detection of pneumonia pathogens in a densely colonised population

**DOI:** 10.15172/pneu.2012.1/209

**Published:** 2012-07-24

**Authors:** Jana Y. R. Lai, Michael J. Binks, Mirjam Kaestli, Amanda J. Leach, Heidi C. Smith-Vaughan

**Affiliations:** 13 Menzies School of Health Research, Charles Darwin University, Northern Territory, Australia; 23 Child Health Division, Menzies School of Health Research, Royal Darwin Hospital, Building 58, Darwin, 0810 Australia

**Keywords:** pneumonia, quantitative real-time PCR, *S. pneumoniae*, *H. influenzae*, serum

## Abstract

Molecular methods offer improvement in the detection of causative pneumonia pathogens, but there are concerns of false positive results. Here we validate quantitative real-time PCR (qPCR) assays for the detection of *Streptococcus pneumoniae* and *Haemophilus influenzae* in: (a) spiked serum samples and (b) in matched serum and nasopharyngeal swabs from a population of Indigenous Australian children without pneumonia, but with a high nasopharyngeal carriage prevalence of *S. pneumoniae* and *H. influenzae*. Matched sera and nasopharyngeal swabs were selected from Indigenous children less than 5 years of age without a diagnosis of pneumonia. Specimens were assayed by qPCR targeting the *lytA* and *glpQ* genes from *S. pneumoniae* and *H. influenzae*, respectively. Using qPCR, neither *S. pneumoniae* nor *H. influenzae* DNA was detected in serum samples, even after concentration of serum DNA. In matched nasopharyngeal swabs, bacterial load was high with up to 106 cells/ml detected by qPCR. In this cohort of children with a high nasopharyngeal carriage, prevalence and bacterial load of pneumonia pathogens, qPCR on sera would not have produced a false pneumonia diagnosis. Thus, qPCR analysis of sera appears to be an appropriate method to aid aetiological diagnosis of pneumonia in this population.

## 1. Introduction

Bacterial pneumonia claims the lives of 10.8 million children annually [1], yet the diagnosis of both bacteraemic and non-bacteraemic pneumonia remains a challenge. Definitive diagnosis of bacteraemic pneumonia is difficult due to the reliance on culture-based methods [2]. Currently, blood culture detects less than 10% of pneumococcal pneumonia cases in children [3].

PCR methods have greatly improved the sensitivity of pneumococcus detection from clinical specimens. The advantages of quantitative realtime PCR (qPCR) processes are its speed, limit of detection and ability to process large numbers of specimens. These features make qPCR an appealing tool for the diagnosis of disease.

Several studies have specifically used qPCR to detect *Streptococcus pneumoniae* and *Haemophilus influenzae*. qPCR of the *lyt*A gene has demonstrated a sensitivity of between 47% to 100%, and a specificity of 80% to 100% across studies investigating serum, whole blood, middle ear fluid and cerebrospinal fluid [3–6]. One study by Rello et al. (2009) was able to correlate *S. pneumoniae* bacterial loads in whole blood ≥ 10^3^ copies/ml with poorer clinical outcomes [7]. However, there are concerns over false positive results with the enhanced sensitivity of molecular methods, stemming from issues with the pneumolysin-based PCR for detection of *S. pneumoniae* detecting other streptococci [8]. For our population, false positive results are of great concern due to the dense nasopharyngea colonisation in Indigenous children which has seen assays such as the rapid antigen-based assay BinaxNOW (Inverness Medical Innovations Inc, USA) not considered for use due to the false-positives in children who were colonised with *S. pneumoniae* [9].

The first aim of this study was to adapt and optimise published *S. pneumoniae* and *H. influenzae* DNA extraction and qPCR methods [10] for detection of these pathogens in serum samples. The qPCR sensitivity and specificity was determined using spiked serum samples. The second aim of the study was to determine if the sensitivity of a qPCR assay would detect false positives in serum from Indigenous children with varying density of nasopharyngeal colonisation. This was determined through testing of matched serum and nasopharyngeal swabs (NPS) from Indigenous children less than 5 years of age, without pneumonia, but with varying density of nasopharyngeal colonisation.

## 2. Methods and results

### 2.1. Method

To determine the most sensitive DNA extraction method, serum samples from healthy volunteers were spiked with reference strains of *S. pneumoniae* (ATCC 49619) and *H. influenzae* (ATCC 49274) at approximately 107 colony forming units/ml or to 0.6 optical density. DNA was extracted from the spiked samples using three commercially available kits; the UltraClean DNA BloodSpin Kit (MO BIO Laboratories, Inc., USA), the Wizard SV Genomic DNA Purification System (Promega Corporation, USA) and the QIAamp DNA Blood Mini Kit (QIAGEN, Germany). The blood protocol was followed for each kit. The QIAamp DNA Blood Mini Kit consistently yielded the greatest quantity of bacterial DNA when an optimised serum starting volume and DNA elution volume of 100 µl were used. Testing of pneumococcal serotypes with varying degrees of capsule expression (serotype 3, 19F and 14) did not affect the results.

1, 2 and 4 µl of each extraction was subsequently used as template in *S. pneumoniae* and *H. influenzae* qPCR assays [10], targeting *lytA* [5] and *glpQ* [11] genes, respectively. Each qPCR included 5 quantitative DNA standards with an unspiked serum extraction as a negative control. *S. pneumoniae* standards ranged from 8.92 × 10^4^ to 8.92 genome copies and *H. influenzae* standards ranged from 9.91 × 10^4^ to 9.91 genome copies. 2 µl of template enhanced sensitivity but inhibition was evident with 4 µl of template. In summary, using the QIAamp DNA Blood Mini Kit of elution volume and 2 µl of extraction as template for the qPCR enabled detection of 446 and 496 cellular equivalents of *S. pneumoniae* and *H. influenzae*, respectively. No qPCR inhibition was evident with these conditions.

### 2.2. Clinical evaluation

For clinical validation of this method it was imperative to rule out the detection of serum positives during nasopharyngeal carriage. Thirtynine serum samples (stored at −80°C) and 30 corresponding NPS (stored in 1ml in skim-milk tryptone glucose glycerol broth [12] at −80°C) from Indigenous children under the age of 5 years were selected based on microbiologically documented positive carriage for *S. pneumoniae* or *H. influenzae* or carriage of both bacteria. Briefly, 100 µl of serum was extracted and quantified as above and 100 µl of corresponding NPS was extracted and quantified as reported previously [10]. All sera were negative for both *S. pneumoniae* and *H. influenzae* as determined by qPCR while over >105 bacterial copies of both bacteria were found in at least 22 culture positive NPS (Table 1).

**Table 1:** qPCR detection of *S. pneumoniae* and *H. influenzae* in clinical samples

		*S. pneumoniae*		*H. influenzae*	
	N	qPCR Positive	Cells/ml (median)	qPCR Positive	Cells/ml (median)
Serum	39	0	-	0	-
NPS	30	27 (22 ^b^)	1.01×10^6 a^	25 (24 ^b^)	6.61×10^5 a^

^a^For *S. pneumoniae* and *H. influenzae* 95% Confidence Intervals (CI) are 5.53×10^5^–6.99×10^6^ and 6.93×10^5^–3.46×10^6^, respectively. 95% CI are percentile bootstrap estimates. Statistical analysis was done using Stata IC Version 11 (StataCorp, USA).

^b^Number positive by culture

To further ensure the absence of detectable bacteria in the blood of high carriage sera, ethanol precipitation was performed on all DNA extracts to increase template concentration five-fold. No additional positives were exposed.

### 2.3 Ethical approval

Ethical approval was granted from the Human Research Ethics Committee of Northern Territory Department of Health and Menzies School of Health Research with reference number 08/84.

## 3. Discussion

Detection and diagnosis of bacterial pathogens in paediatric populations is limited by small blood volume and a slow, insensitive gold standard (blood culture). While serum qPCR of adult patients with community-acquired pneumonia has been shown to detect as low as 8 cellular equivalents per ml, larger blood volumes are used [7]. In this study, using paediatric volumes of serum, we were able to detect to 446 and 496 cellular equivalents of *S. pneumoniae* and *H. influenzae* per 100 µl. This equates to a blood infection of approximately 10^4^ cells/ml. These qPCR assays can be run concurrently and take 5 hours from receipt of sample to final result.

## 4. Conclusion

qPCR of *S. pneumoniae* or *H. influenzae* DNA in serum is rapid, reproducible and appropriate for children in populations experiencing dense nasopharyngeal colonisation of the target pathogens.
